# An Effective Phytoconstituent Aconitine: A Realistic Approach for the Treatment of Trigeminal Neuralgia

**DOI:** 10.1155/2021/6676063

**Published:** 2021-04-14

**Authors:** Dilek Çankal, Esra Küpeli Akkol, Yeliz Kılınç, Mert İlhan, Raffaele Capasso

**Affiliations:** ^1^Department of Oral and Maxillofacial Surgery, Faculty of Dentistry, Gazi University, 06490 Ankara, Turkey; ^2^Department of Pharmacognosy, Faculty of Pharmacy, Gazi University, 06330 Ankara, Turkey; ^3^Department of Pharmacognosy, Faculty of Pharmacy, Van Yüzüncü Yıl University, 65080 Van, Turkey; ^4^Department of Agricultural Sciences, University of Naples Federico II, 80055 Portici Naples, Italy

## Abstract

Trigeminal neuralgia pain remains a challenge to treat. Natural compounds may be promising options for relieving pain. This study was aimed at investigating the effects of aconitine in a rat model of trigeminal neuralgia pain. Infraorbital nerve chronic constriction injury was performed in adult Wistar Albino rats. After the neuropathic pain developed, the rats were assigned to one of the treatment groups: carbamazepine 40 or 80 mg/kg; aconitine 0.25, 0.50, or 0.75 mg/kg; or saline injection (control group). Behavioral testing with von Frey filaments and the rotarod test were carried out before the surgical procedure and on the 24th to 29th postoperative days. Following the completion of tests, ipsilateral and contralateral spinal cords were harvested for Western blot analyses to assess NR-1 protein expression. ANOVA followed by Mann-Whitney *U* test was performed for the statistical analyses. *P* values of <0.05 were considered significant. Aconitine significantly reduced mechanical sensitivity in a dose-dependent manner. A significant reduction in motor coordination was noted for the higher doses of aconitine which was similar with the 40 and 80 mg/kg doses of carbamazepine. NR-1 expression was reduced in the ipsilateral spinal cord, whereas no significant difference was noted between the groups in the expression of NR-1 in the contralateral spinal cord. Aconitine had a significant pain relieving effect, which was similar to carbamazepine, in a dose-dependent manner. Aconitine may be an alternative pharmacological agent for the control of trigeminal neuralgia pain.

## 1. Introduction

Trigeminal neuralgia (TN) is a neuropathic pain condition in the distribution of one or more branches of the trigeminal nerve [1]. TN is defined by the 3rd International Classification of Headache Disorders (ICHD-3) as “a sudden, recurrent unilateral brief electric shock-like stabbing, or shooting pain lasting between a fraction of a second to 2 minutes” [2, 3]. The attacks may occur spontaneously or be triggered by innocuous stimuli including talking, chewing, brushing, and touching [4, 5]. However, pain-free periods exist between the attacks [6]. According to the new classification of the International Association for the Study of Pain, TN has been classified into classical, occurring as a result of neurovascular compression; idiopathic, when no etiologic factor is evident; and secondary, caused by tumors, infections, multiple sclerosis, trauma, etc. [3, 7]. The incidence rate is estimated to be 4-5 per 100,000, being more common in females than males and those above 40 years of age [4, 6].

Studies have shown that medication is efficacious in most of the patients, and pharmacotherapy is considered as the first-line treatment for TN [4, 8–10]. However, surgical treatment should be considered in the presence of structural lesions and for cases refractory to medications. Conventional analgesics and anti-inflammatory medications do not alleviate TN pain, whereas antiepileptics are usually beneficial [11]. Carbamazepine has been recognized as the first-line medication for the initial medical treatment of TN in many clinical guidelines [7, 12]. Carbamazepine has been reported to provide a 100% pain relief in 70% of patients [13]. However, patients usually experience side effects including drowsiness, dizziness, constipation, nausea, and ataxia. Less common but more severe side effects include rashes, leucopenia, abnormal liver function, aplastic anemia, and hyponatremia [3]. Numerous drug interactions with carbamazepine have also been mentioned in the literature [14]. This suggests a need to evaluate alternative pharmacologic agents with less side effects that can be used safely in all patient groups in the treatment of TN.

Natural compounds may have a therapeutic value for neuropathic pain relief. Since ancient times, preparations of various *Aconitum* L. species, which belong to the Ranunculaceae family, have been widely used as remedies in Eastern medicine [15]. *Aconitum* species comprises over 300 species which grow naturally all over the world [16]. Several types of alkaloids have been extracted from different *Aconitum* species [17] . Aconitine, the main *Aconitum* alkaloid, and other related alkaloids have been employed especially in traditional Chinese medicine and Ayurvedic medicine for analgesic, anti-inflammatory, antirheumatic, and neurological indications [15, 17]). The analgesic effects of the Aconitum species have been demonstrated in animal models of inflammatory and neuropathic pain [18]. The extract obtained from *Aconitum* species was applied externally for the treatment of TN, lumbago, sciatica, arthritis, and gout due to its anesthetic properties [19].

The effects of systemic administration of aconitine on TN remain unknown. In this study, we investigated the effects of aconitine on TN in the rat chronic constriction injury (CCI) of an infraorbital nerve (ION) model.

## 2. Materials and Methods

### 2.1. Experimental Animals

The rats used in this study were obtained from the Kobay Animals Laboratory. The study protocol was approved by the ethics committee of the Kobay Animals Laboratory (Approval Number: 234). A total of 72 male Wistar Albino rats with a mean weight of 180-200 gr were used in the present study. The animals were kept in standard plastic cages in a room with a humidity of 40-45% and temperature at 21-24°C with 12/12 h light/dark cycles, with food and water ad libitum.

### 2.2. Experimental Protocol

Seventy-two rats weighing 180-200 g were included in the study. At baseline, all rats were tested for motor coordination and subjected to behavioral testing for tactile allodynia. All rats underwent infraorbital nerve chronic constriction injury. Following the injury and neuropathic pain development, the rats were divided into 6 groups (12 rats each). Two groups were treated with different doses of carbamazepine (40 and 80 mg/kg) intraperitoneally, 3 groups were treated with different doses of aconitine (0.25, 0.50, and 0.75 mg/kg) intraorally. One group served as the control and received only saline injection intraperitoneally. The order in which these six different treatments were tested differed among animals; a Latin square design was used to ensure that each day all six treatments were tested. Aconitine was applied at the dose of 0.25, 0.50, and 0.75 mg/kg by gastric gavage. As a reference drug, carbamazepine was applied at the dose of 40 and 80 mg/kg, intraperitoneally. Those doses were selected according to Barros and Leite and Carroll et al. [20, 21]. The medications were administered during six consecutive postoperative days (i.e., 24th to 29th day) 30 minutes before the start of the behavioral testing. The doses were determined based on the doses described in the literature [22]. After the completion of tests, 36 rats were used for Western blot analysis and the spinal cords were harvested under general anesthesia.

### 2.3. Surgical Protocol

The CCI-ION was performed according to the method as described previously [23]. Briefly, rats were anesthetized with intramuscular administration of the combination of 1 mL ketamine (50 mg/mL) and 1 mL xylazine (20 mg/mL). The incision was carried out at the junction between the nasal bone and the zygomatic arch. The ION was exposed by means of blunt dissection of the muscle. Following dissection of the ION from the adjacent tissues, two ligations were done with chromic catgut around the nerve. The incision was closed with silk sutures.

### 2.4. Behavioral Testing

Rats were tested at baseline and 24th-29th days after surgery. On the day before the surgery, before the first actual stimulation session, the rats were allowed to adapt to the observation cage and to the testing environment for 3 h. During this period, the experimenter reached slowly into the cage to touch the walls of the cage with a plastic rod, similar to the ones on which the von Frey filaments are mounted. After the rats were habituated to the reaching movements, the series of mechanical stimulations were started [24]. The tests performed 30 minutes after the administration of the medications. Acclimation and testing were carried out in a darkened room with a light provided by a 60 W red light bulb suspended 1 m above the center of the test area with a 45 dB background noise. As described previously [25], the mechanical sensitivity was tested by applying graded forces (0.015, 0.127, 0.217, 0.745, and 2.150 g) with von Frey filaments (Pressure Aesthesiometer®, Stoelting, Chicago, IL). The filaments were applied within the ION territory. The stimuli were administered unilaterally before surgery and bilaterally after surgery, with an increasing intensity. The scoring, based on the previously validated scale, was performed to assess the response of the rats for each stimulus [25]. The scoring was as follows: no response = 0; detection = 1; detection and withdrawal = 2; detection, withdrawal, and escape or attacking movements = 3; and same as in response 3 but with extended ipsilateral facial grooming > 3 strokes = 4. During the von Frey tests, animals were immobile.

### 2.5. Rotarod Test

A rotating rod was used to investigate the effects of the drugs on motor coordination, as previously described [26]. The rotarod speed was calibrated at 16 rpm with the maximum time spent with the rod set at 180 s. The animals were exercised two or three times on two separate days for habituation before the test. The basal response was evaluated on the 14th postoperative day.

### 2.6. Western Blot Analysis

Following the last behavior testing, the spinal cords were removed under general anesthesia and separated into ipsilateral and contralateral parts. The samples were stored at -80°C for Western Blot analysis.

In scientific studies, it has been shown that carbamazepine inhibits the increase of intracellular free calcium induced by glycine and NMDA (N-methyl-D-aspartate) in granular cells of the rat cerebellum and affects pain by blocking the release of endogenous glutamate [27]. In the present study, the NMDA values were evaluated. The effects of glycine transporter inhibitors on NR-1 protein expression were investigated as previously described [28].

### 2.7. Statistical Evaluation

Statistical analyses were performed using GraphPad Prism (version 6.0). *P* values of <0.05 were considered significant: ^∗^*P* < 0.05, ^∗∗^*P* < 0.01, ^∗∗∗^*P* < 0.001, and ^∗∗∗∗^*P* < 0.0001. One-way ANOVA preceded by a Bonferroni test (95% confidence interval) was carried out when comparing three or more groups. For the statistical evaluation in the behavioral and rotarod test, each time was compared within itself. All results reported statistical mean ± standard error of the mean (SEM).

## 3. Results

### 3.1. Behavioral Testing

**Figure 1:**
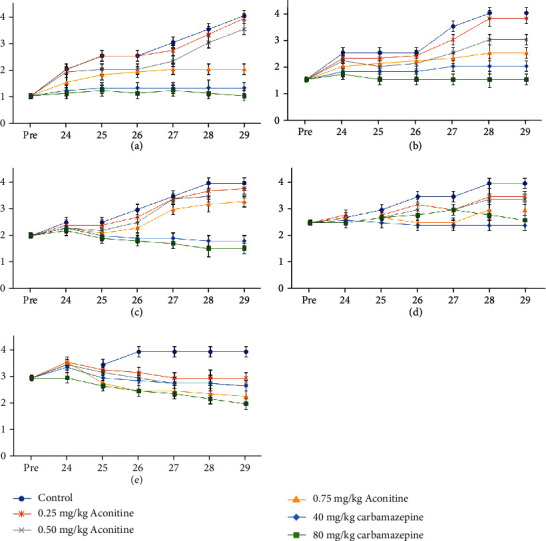
Response levels given to ipsilateral stimulations in behavioral tests with von Frey filaments with (a) 0.015, (b) 0.127, (c) 0.217, (d) 0.745, and (e) 2.150 g bond strength (*x* axis represents days, *y* axis represents score).

**Figure 2:**
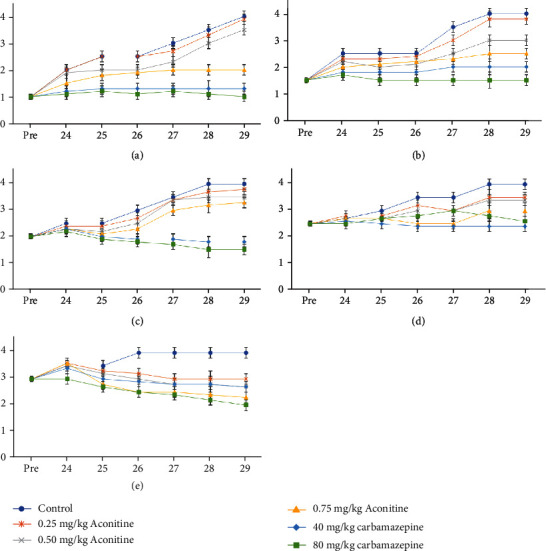
Response levels given to contralateral stimulations in behavioral tests with von Frey filaments with (a) 0.015, (b) 0.127, (c) 0.217, (d) 0.745, and (e) 2.150 g bond strength (*x* axis represents days, *y* axis represents score).

A significant decrease toward mechanical stimuli with von Frey filaments was found in responses to ipsilateral and contralateral stimulations performed at all doses of aconitine and carbamazepine (Figures 1 and 2). The antiallodynic effect was in a dose-dependent manner for both carbamazepine and aconitine groups. 80 mg carbamazepine and 0.75 mg aconitine had the most significant effect among the groups.

### 3.2. Rotarod Test

**Figure 3:**
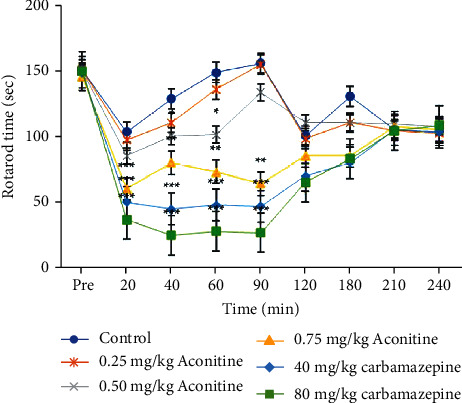
Effects of test samples on motor coordination in rotarod test.

No significant effect was found for the 0.25 mg/kg aconitine group on the motor performance of the rats when compared to the control group. However, aconitine administered at 0.50 and 0.75 mg/kg doses showed a significant antiallodynic effect at the 20th, 40th, 60th, and 90th minutes by significantly impairing motor coordination (Figure 3). Carbamazepine had a significant effect on motor coordination similar to aconitine at all doses.

### 3.3. Western Blot Analysis

**Figure 4:**
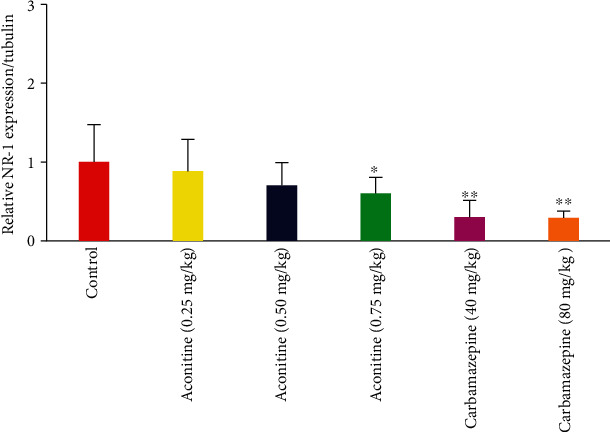
Effects of test samples on NR-1 expression in ipsilateral spinal cord in Western blot test.

It was demonstrated that aconitine reduced NR-1 expression in the ipsilateral spinal cord at all doses, but only 0.75 mg/kg of aconitine was found to be statistically significant (Figure 4).

**Figure 5:**
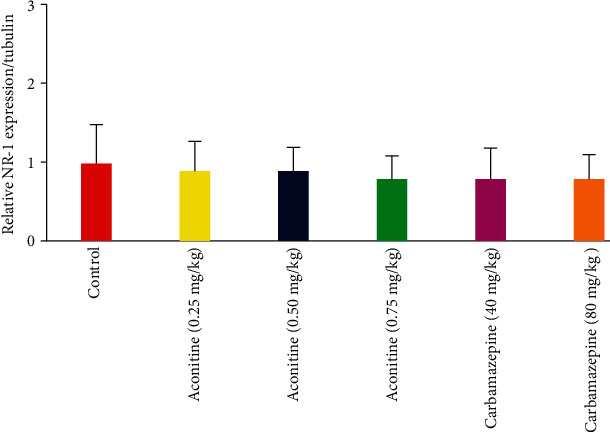
Effects of test specimens on NR-1 expression in the contralateral spinal cord in the Western blot test.

However, it was concluded that there was no significant difference between the groups in the expression of NR-1 in the contralateral spinal cord (Figure 5).

## 4. Discussion

Among different neuropathic pain conditions, TN is the most commonly encountered neuralgic cause of facial pain [5]. Patients with facial pain frequently consult their dental practitioner and may receive further dental evaluation and treatment before they are referred to a neurologist [29]. The diagnosis for TN is still based on clinical findings, clinical history, and examination due to the lack of objective testing methods [30, 31]. TN is characterized by periods of partial or complete remission and spontaneous recovery is rare [32]. Currently, there is no cure for TN, and the management of the condition remains a challenge, often resulting in misdiagnosis [31].

The pain experienced with TN has a significant impairment on the quality of life with suboptimal outcomes due to the lack of efficacy and poor tolerability of current medical therapies [33, 34]. Recent clinical studies have reported that patients present different levels of depression and anxiety [35, 36]. High doses are usually required for sufficient pain relief, and the patients experience distressing side effects and other health issues [2].

TN is a chronic peripheral neuropathic pain condition, and neuropathic pain has a poor response to conventional therapeutic methods and standard doses of opioid analgesics [37, 38]. Antiepileptic drugs are used for the management of TN pain due to the similarities between the biochemical and molecular mechanisms demonstrated in some experimental epilepsy and neuropathic pain models [37]. In recent years, little progress has been made in the treatment of TN. Currently, there is no specific drug for TN [33]. Of the commonly used antiepileptic agents in TN, carbamazepine is the first-choice medical therapy and has a significant effect in providing TN pain relief [39]. The mechanism of action for carbamazepine is the blockade of the voltage gated sodium channel in a frequency-dependent manner, resulting in the stabilization of hyperexcited neural membranes and in the inhibition of repetitive firing. However, long-term treatment with carbamazepine has been associated with serious side effects, causing interruption of treatment or dosage reduction [40].

Medicinal plants have been used in traditional medicine for centuries, and some of these plants possess analgesic properties. These plants may provide an alternative option for the management of pain [41]. Among these plants, the biological properties of *Aconitum* species have been widely known [42]. The *Aconitum* species have been employed as herbal medicine in Eastern traditional medicine for their anti-inflammatory and analgesic effects. The pharmacologic effects are partly associated with aconitine [42, 43]. Aconitine, being the main *Aconitum* alkaloid, has been known as a potent neurotoxin whereas it also provides antinociception in rodent models [44].

The effects of aconitine on TN were evaluated in an experimental neuropathic pain model (CCI of the ION) in the present study. CCI of the ION was described in 1994 and has been used as a major tool to investigate trigeminal neuropathic pain [25, 45]. This pain model has the advantage of inducing a pure sensory nerve injury leading to painful sensory disturbances in the ION territory. Animals subjected to CCI-ION exhibit behavioral alterations consistent with clinical symptoms of trigeminal nerve injury. The CCI-ION model was selected as this model could finely represent the clinical situation of TN pain [45]. To the authors' knowledge, the present study constitutes the first investigation of the effects of aconitine on TN. The results clearly demonstrated that aconitine could produce a significant antiallodynic effect in a rat CCI-ION neuropathic pain model.

The voltage-dependent sodium channel is known as a primary target for the analgesic effects of *Aconitum* species [18]. Aconitine acts by suppressing the inactivation of voltage-dependent Na+ channels by binding to the neurotoxin binding site 2 of the *α*-subunit of the channel protein [46]. Due to the prolonged Na^+^ channel activation, the cells are depolarized by a sustained Na^+^ influx finally leading to inexcitability [47]. It is one of the most powerful modifiers of the voltage-dependent Na^+^ channel [46]. The antiallodynic effect of aconitine demonstrated in this study could be the result of its activity on the sodium channel of neurons on the spinal cord.

Previous experimental studies have reported the antinociceptive effects of aconitine and other related alkaloids of the *Aconitum* species in different pain models. The processed *Aconiti* tuber has been reported to relieve neuropathic pain in the rat chronic constriction injury model [48]. Bulleyaconitine A was found to be effective for treating chronic pain, including back pain and neuropathic pain in patients with minimal side effects [41]. A case series study on 12 postherpetic neuralgia patients showed an improvement in pain levels with the application of *Aconitum* preparations [49]. It has also been reported as a promising agent for diabetic peripheral neuropathic pain [50]. Lappaconitine, another alkaloid extracted from the *Aconitum* species, produced significant pain relief in a rat model of neuropathic pain induced by CCI of the sciatic nerve. The inhibition on the nociceptive behaviors was mainly based on the decreased expression of the P2X3 receptors in the DRG neurons [51].

Aconitine did not induce any adverse effect influencing the pain thresholds. These findings suggest that treatment with aconitine significantly attenuated neuropathic pain in response to nerve injury. Additionally, the range of the response score was 2-4 in both ipsilateral and contralateral stimulations with 0.745 and 2.150 g bond strength. On the other hand, the range of the response score was 1-4 in both ipsilateral and contralateral stimulations with 0.015, 0.127, and 0.217 g bond strength. The aconitine administration at the dose of 0.75 mg/kg remarkably decreased especially on the 28th and 29th days with 0.015 g bond strength. Besides, the administration of aconitine at the doses of 0.25, 0.50, and 0.75 mg/kg remarkably decreased on the 26th, 27th, 28th, and 29th days with 2.150 g bond strength in both behavioral tests. In the present study, it was determined that the response scores have been increased during the 24-day postoperative period. However, especially during the last 4 days, the response scores have been increased in the control groups. With the 0.217 g bond strength, the carbamazepine administration at the doses of 40 and 80 mg/kg remarkably decreased the response scores between the 26th and 29th days.

Sedation and attenuated motor coordination are well-recognized side effects of antiepileptic drugs [37, 52]. The 0.25 mg/kg dose of aconitine had no effect on motor coordination. However, the higher doses of aconitine (0.50 mg/kg and 0.75 mg/kg) resulted with a significant reduction in motor coordination, which was similar with the 40 and 80 mg/kg doses of carbamazepine (20th, 40th, 60th, and 90th minutes). The rats were able to tolerate the aconitine doses and remain on the rotating rod.

NR-1, the glycine-binding subunit of the excitatory NMDA receptor, has a critical role in the development of neuropathic pain [28]. However limited information has been discussed in the literature regarding the role of the NMDA receptor in the antiallodynic effect provided by aconitine. A significant reduction of NR-1 was observed in the ipsilateral spinal cord for 0.75 mg aconitine. This novel finding suggests that aconitine has a crucial effect on NMDA receptors.

Although the present study has provided an important documentation on the rat model following CCI-ION, this model has some limitations requiring further study. Rats with CCI-ION exhibit pain-related behavioral abnormalities and mechanical allodynia [23, 25, 45]. However spontaneous chronic pain mechanisms may be different from experimental models of evoked pain [53], which may not correlate with the clinical findings of TN. Design of different experimental models and examination of chronic pain mechanisms should be included in future studies.

## 5. Conclusions

Our experimental results indicate that aconitine significantly attenuated TN pain, dose dependently, in a rat CCI-ION neuropathic pain model. These pain-relieving effects were probably mediated via NMDA receptor mechanisms. The pain-relieving effect was similar with carbamazepine. Up till now, carbamazepine has been the golden standard for the treatment of TN pain despite severe side effects in the long term. Aconitine appears to be a promising pharmacological agent for the control of TN pain.

## Data Availability

The data used to support the findings of this study are included within the article.

## References

[1] Ghurye S., McMillan R. (2017). Orofacial pain - an update on diagnosis and management. *British Dental Journal*.

[2] Maarbjerg S., Di Stefano G., Bendtsen L., Cruccu G. (2017). Trigeminal neuralgia – diagnosis and treatment. *Cephalalgia*.

[3] Mupparapu M., Ko E., Omolehinwa T. T., Chhabra A. (2020). Neurologic disorders of the maxillofacial region. *Dental Clinics of North America*.

[4] Sathasivam H. P., Ismail S., Ahmad A. R. (2017). Trigeminal neuralgia: a retrospective multicentre study of 320 Asian patients. *Oral Surgery, Oral Medicine, Oral Pathology, Oral Radiology*.

[5] Sahoo N. K., Thakral A., Deb P., Roy I. D. (2020). Histopathological evaluation of inferior alveolar neurovascular bundle in cases of trigeminal neuralgia. *Journal of Oral and Maxillofacial Surgery*.

[6] Balasubramaniam R., Klasser G. D. (2014). Orofacial pain syndromes: evaluation and management. *The Medical Clinics of North America*.

[7] Cruccu G., Finnerup N. B., Jensen T. S. (2016). Trigeminal neuralgia. *Neurology*.

[8] Watson C. P. (2004). Management issues of neuropathic trigeminal pain from a medical perspective. *Journal of Orofacial Pain*.

[9] Gronseth G., Cruccu G., Alksne J. (2008). Practice parameter: the diagnostic evaluation and treatment of trigeminal neuralgia (an evidence-based review): report of the Quality Standards Subcommittee of the American Academy of Neurology and the European Federation of Neurological Societies. *Neurology*.

[10] McLeod N. M., Tekeli K. M., Cheriyan J. J. (2009). Trigeminal neuralgia: assessment and management by oral and maxillofacial surgeons in the United Kingdom. *The British Journal of Oral & Maxillofacial Surgery*.

[11] Wright E., Evans J. (2014). Oral pre-trigeminal neuralgia pain: clinical differential diagnosis and descriptive study results. *The Journal of Cranio-Mandibular Practice*.

[12] Bendtsen L., Zakrzewska J. M., Abbott J. (2019). European Academy of Neurology guideline on trigeminal neuralgia. *European Journal of Neurology*.

[13] Wiffen P. J., Derry S., Moore R. A., McQuay H. J. (2011). Carbamazepine for acute and chronic pain in adults. *Cochrane Database of Systematic Reviews*.

[14] Jorns T. P., Zakrzewska J. M. (2007). Evidence-based approach to the medical management of trigeminal neuralgia. *British Journal of Neurosurgery*.

[15] Xu H., Arita H., Hayashida M., Zhang L., Sekiyama H., Hanaoka K. (2006). Pain-relieving effects of processed Aconiti tuber in CCI-neuropathic rats. *Journal of Ethnopharmacology*.

[16] Singhuber J., Zhu M., Prinz S., Kopp B. (2009). Aconitum in traditional Chinese medicine --a valuable drug or an unpredictable risk?. *Journal of Ethnopharmacology*.

[17] Tai C. J., el-Shazly M., Wu T. Y. (2015). Clinical aspects of *Aconitum* preparations. *Planta Medica*.

[18] Yang J., Park K. S., Yoon J. J., Bae H. B., Yoon M. H., Choi J. I. (2016). Anti-allodynic effect of intrathecal processed Aconitum jaluense is associated with the inhibition of microglial activation and P2X7 receptor expression in spinal cord. *BMC Complementary and Alternative Medicine*.

[19] Povšnar M., Koželj G., Kreft S., Lumpert M. (2017). Rare tradition of the folk medicinal use of Aconitum spp. is kept alive in Solčavsko, Slovenia. *Journal of Ethnobiology and Ethnomedicine*.

[20] Barros H. M. T., Leite J. R. (1987). The effects of carbamazepine on two animal models of depression. *Psychopharmacology*.

[21] Carroll M. E., Lac S. T., Asencio M., Halikas J. A., Kragh R. (1990). Effects of carbamazepine on self-administration of intravenously delivered cocaine in rats. *Pharmacology, Biochemistry, and Behavior*.

[22] Gao X., Hu J., Zhang X., Zuo Y., Wang Y., Zhu S. (2020). Research progress of aconitine toxicity and forensic analysis of aconitine poisoning. *Forensic Sciences Research*.

[23] Liu C. Y., Lu Z. Y., Li N., Yu L. H., Zhao Y. F., Ma B. (2015). The role of large-conductance, calcium-activated potassium channels in a rat model of trigeminal neuropathic pain. *Cephalalgia*.

[24] Idänpään-Heikkilä J. J., Guilbaud G. (1999). Pharmacological studies on a rat model of trigeminal neuropathic pain: baclofen, but not carbamazepine, morphine or tricyclic antidepressants, attenuates the allodynia-like behaviour. *Pain*.

[25] Vos B. P., Strassman A. M., Maciewicz R. J. (1994). Behavioral evidence of trigeminal neuropathic pain following chronic constriction injury to the rat's infraorbital nerve. *The Journal of Neuroscience*.

[26] Jeon H. J., Han S. R., Park M. K., Yang K. Y., Bae Y. C., Ahn D. K. (2012). A novel trigeminal neuropathic pain model: compression of the trigeminal nerve root produces prolonged nociception in rats. *Progress in Neuro-Psychopharmacology & Biological Psychiatry*.

[27] Davies J. A. (1995). Mechanisms of action of antiepileptic drugs. *Seizure*.

[28] Barthel F., Urban A., Schlösser L. (2014). Long-term application of glycine transporter inhibitors acts antineuropathic and modulates spinal N-methyl-d-aspartate receptor subunit NR-1 expression in rats. *Anesthesiology*.

[29] Von Eckardstein K. L., Keil M., Rohde V. (2015). Unnecessary dental procedures as a consequence of trigeminal neuralgia. *Neurosurgical Review*.

[30] Oomens M. A., Forouzanfar T. (2015). Pharmaceutical management of trigeminal neuralgia in the elderly. *Drugs & Aging*.

[31] Allsop M. J., Twiddy M., Grant H. (2015). Diagnosis, medication, and surgical management for patients with trigeminal neuralgia: a qualitative study. *Acta Neurochirurgica*.

[32] Di Stefano G., Truini A. (2017). Pharmacological treatment of trigeminal neuralgia. *Expert Review of Neurotherapeutics*.

[33] Zakrzewska J. M., Wu N., Lee J., Werneburg B., Hoffman D., Liu Y. (2018). Characterizing treatment utilization patterns for trigeminal neuralgia in the United States. *The Clinical Journal of Pain*.

[34] Araya E. I., Claudino R. F., Piovesan E. J., Chichorro J. G. (2020). Trigeminal neuralgia: basic and clinical aspects. *Current Neuropharmacology*.

[35] Melek L. N., Devine M., Renton T. (2018). The psychosocial impact of orofacial pain in trigeminal neuralgia patients: a systematic review. *International Journal of Oral and Maxillofacial Surgery*.

[36] Melek L. N., Smith J. G., Karamat A., Renton T. (2019). Comparison of the neuropathic pain symptoms and psychosocial impacts of trigeminal neuralgia and painful posttraumatic trigeminal neuropathy. *Journal of Oral & Facial Pain and Headache*.

[37] Spina E., Perugi G. (2004). Antiepileptic drugs: indications other than epilepsy. *Epileptic Disorders*.

[38] Szok D., Tajti J., Nyári A., Vécsei L., Trojano L. (2019). Therapeutic approaches for peripheral and central neuropathic pain. *Behavioural Neurology*.

[39] Zakrzewska J. M., Linskey M. E. (2014). Trigeminal neuralgia. *BMJ Clinical Evidence*.

[40] Di Stefano G., Truini A., Cruccu G. (2018). Current and innovative pharmacological options to treat typical and atypical trigeminal neuralgia. *Drugs*.

[41] Luo Y., Wang C. Z., Sawadogo R., Tan T., Yuan C. S. (2020). Effects of herbal medicines on pain management. *The American Journal of Chinese Medicine*.

[42] Tarbe M., de Pomyers H., Mugnier L. (2017). Gram-scale purification of aconitine and identification of lappaconitine in Aconitum karacolicum. *Fitoterapia*.

[43] Li M., He J., Jiang L. L. (2013). The anti-arthritic effects of Aconitum vilmorinianum, a folk herbal medicine in Southwestern China. *Journal of Ethnopharmacology*.

[44] Yamanaka H., Doi A., Ishibashi H., Akaike N. (2002). Aconitine facilitates spontaneous transmitter release at rat ventromedial hypothalamic neurons. *British Journal of Pharmacology*.

[45] Ding W., You Z., Shen S. (2017). An improved rodent model of trigeminal neuropathic pain by unilateral chronic constriction injury of distal infraorbital nerve. *The Journal of Pain*.

[46] A. Ameri A. (1998). The effects of Aconitum alkaloids on the central nervous system. *Progress in Neurobiology*.

[47] Friese J., Gleitz J., Gutser U. T. (1997). Aconitum sp. alkaloids: the modulation of voltage-dependent Na^+^ channels, toxicity and antinociceptive properties. *European Journal of Pharmacology*.

[48] Hao D. C., Gu X. J., Xiao P. G. (2015). *Medicinal plants: chemistry, biology and omics*.

[49] Nakanishi M., Arimitsu J., Kageyama M. (2012). Efficacy of traditional Japanese herbal medicines—Keishikajutsubuto (TJ-18) and Bushi-matsu (TJ-3022)—against postherpetic neuralgia aggravated by self-reported cold stimulation: a case series. *Journal of Alternative and Complementary Medicine*.

[50] Feng L., Liu W. K., Deng L., Tian J. X., Tong X. L. (2014). Clinical efficacy of aconitum-containing traditional Chinese medicine for diabetic peripheral neuropathic pain. *The American Journal of Chinese Medicine*.

[51] Ou S., Zhao Y. D., Xiao Z., Wen H. Z., Cui J., Ruan H. Z. (2011). Effect of lappaconitine on neuropathic pain mediated by P2X_3_ receptor in rat dorsal root ganglion. *Neurochemistry International*.

[52] Tentolouris-Piperas V., Lee G., Reading J., O'Keeffe A. G., Zakrzewska J. M., Cregg R. (2018). Adverse effects of anti-epileptics in trigeminal neuralgiform pain. *Acta Neurologica Scandinavica*.

[53] Deseure K., Hans G. (2015). Behavioral study of non-evoked orofacial pain following different types of infraorbital nerve injury in rats. *Physiology & Behavior*.

